# Baculovirus LEF-11 Hijack Host ATPase ATAD3A to Promote Virus Multiplication in *Bombyx mori* cells

**DOI:** 10.1038/srep46187

**Published:** 2017-04-10

**Authors:** Zhan-Qi Dong, Nan Hu, Fei-Fan Dong, Ting-Ting Chen, Ya-Ming Jiang, Peng Chen, Cheng Lu, Min-Hui Pan

**Affiliations:** 1 State Key Laboratory of Silkworm Genome Biology, Southwest University, Chongqing 400716, China; 2 Key Laboratory for Sericulture Functional Genomics and Biotechnology of Agricultural Ministry, Southwest University, Chongqing 400716, China

## Abstract

Research on molecular mechanisms that viruses use to regulate the host apparatus is important in virus infection control and antiviral therapy exploration. Our previous research showed that the *Bombyx mori nucleopolyhedrovirus* (BmNPV) LEF-11 localized to dense regions of the cell nucleus and is required for viral DNA replication. Herein, we examined the mechanism of LEF-11 on BmNPV multiplication and demonstrated that baculovirus LEF-11 interacts with *Bombyx mori* ATAD3A and HSPD1 (HSP60) protein. Furthermore, we showed that LEF-11 has the ability to induce and up-regulate the expression of ATAD3A and HSPD1, phenomena that were both reversed upon knockdown of *lef-11*. Our findings showed that ATAD3A and HSPD1 were necessary and contributed to BmNPV multiplication in *Bombyx mori* cells. Moreover, ATAD3A was found to directly interact with HSPD1. Interestingly, ATAD3A was required for the expression of HSPD1, while the knockdown of HSPD1 had no obvious effect on the expression level of ATAD3A. Taken together, the data presented in the current study demonstrated that baculovirus LEF-11 hijacks the host ATPase family members, ATAD3A and HSPD1, efficiently promote the multiplication of the virus. This study furthers our understanding of how baculovirus modulates energy metabolism of the host and provides a new insight into the molecular mechanisms of antiviral research.

The ability of viruses to regulate the internal environment of the host cells for their replication and multiplication is a well-known feature that is common to many viruses^1^,^2^,^3^. Upon viral entry into host cells, there is often a systemic reduction of host protein and perturbation of metabolic pathways of the host cells, which result in low levels of metabolites required for host transcription and DNA synthesis, thus exploiting the host apparatus and resources for their replication and multiplication^4^. Therefore, the interaction of viral proteins with host factors, and subsequent regulation of cellular mechanisms and modification of the environment of host cells to promote virus replication are of great significance for their multiplication.

Baculoviruses are DNA viruses with double-stranded, circular and large genome^5^,^6^. Baculoviruses have been reported to be infectious to different species of invertebrates, mainly the insects—*Lepidoptera, Dipter*a and *Hymenoptera*^6^,^7^. Over the past few years, studies examining the interaction between baculovirus and host have yielded much insight concerning baculovirus factors and host cell factor^8^,^9^. Baculoviruses have evolved anti-apoptosis genes IAP to inhibit host cell death responses; multifunctional viral cyclin ODV-EC27 appears to block the cell cycle and contributes to virus replication and F-Box protein LEF-7 promotes virus multiplication via modifying host DNA damage response^10^,^11^,^12^. Previous studies have described that upon infection of host cells, many viruses can modulate energy metabolism of the host cell for their multiplication and replication^13^,^14^. Although there are no genes recognized for enzymes involved in energy metabolism in the functional genomics of baculovirus, previous studies have found that the regulation of energy metabolism promotes the baculovirus infection in the silkworm^14^,^15^,^16^. Transcriptome analysis also indicated that many energy metabolism-related pathways of the host cell are involved in baculovirus infection^9^,^16^,^17^. Current research shows that energy metabolism-related pathways involved in baculovirus infection have included glycolysis pathway activities; for example, guanosine triphosphate (GTP) energy during translation, tricarboxylic acids (TCA) cycle synthesis and central energy metabolism^13^,^18^,^19^,^20^,^21^,^22^. However, additional energy metabolism mechanisms involved in baculovirus infection have not been clearly elucidated in insect cells, especially the regulation mechanisms of those metabolism-related factors and proteins in the baculovirus infection.

Baculovirus late expression factors (LEFs) are involved in viral DNA replication and viral gene regulation^23^,^24^,^25^. Baculovirus *lef-11* gene encodes a putative protein with molecular mass of 13.1 KDa^26^. In our previous study, we indicated that *Bombyx mori nucleopolyhedrovirus* (BmNPV) LEF-11 is conserved in all 63 sequenced baculovirus genomes except *Diptera* CuniNPV^23^. We further identified that LEF-11 contains a nuclear localization signal and localizes to viral DNA replication sites in BmNPV infection cells^27^. Additionally, those results showed that the baculovirus LEF-11 and its oligomerization domains were required for viral DNA replication^23^. Although various studies have demonstrated that LEF-11 plays an important role in viral DNA replication, the cellular mechanisms of LEF-11 regulation are largely unknown.

In the present study, in order to analyze the function of LEF-11, we initially identified BmNPV LEF-11 interacting with ATPase family members ATAD3A and HSPD1 (HSP60) of *Bombyx mori* by co-immunoprecipitation (Co-IP) and mass spectrometry analyses. Moreover, results suggest that LEF-11 could directly activate the expression of *BmATAD3A* and *BmHSPD1*, while *lef-11* gene knockout bacmid had diminished functionality as compared to WT bacmid. In addition, we demonstrated that overexpression of ATAD3A and HSPD1 proteins could effectively promote virus replication and multiplication, while knockdown of ATAD3A and HSDP1 significantly inhibited the multiplication of the virus at the cellular level. Besides, we demonstrate that ATAD3A and HSPD1 can directly interact with each other, and the expression of ATAD3A can directly influence the level of HSPD1 expression, but HSPD1 did not have the same function as ATAD3A. Combined, the data presented here indicate that baculovirus LEF-11 has the ability to induce the host ATAD3A and HSPD1 to promote virus multiplication.

## Results

### Identification of LEF-11-associated protein by Co-IP and mass spectrometry

**Figure 1:**
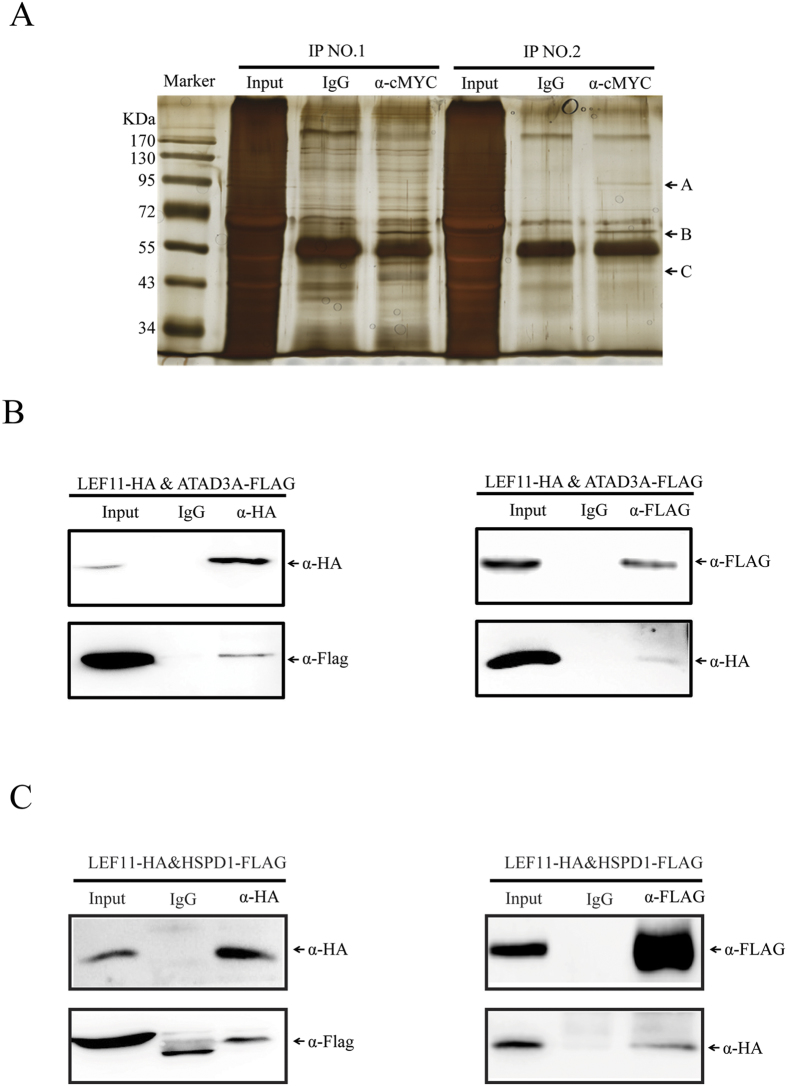
Identification of LEF-11-associated proteins by Co-IP and mass spectrometry. (**A**) Co-IP assays of LEF-11-associated protein analyzed by SDS-PAGE. Marker, protein molecular weight marker; Input, input cell lysates; IgG, IP with control mouse IgG; α-cMYC, IP with anti-cMYC antibody. The specific bands represented by the arrows. IP No.1 and IP No.2 is representative of two repeated experiments. (**B**) Co-immunoprecipitation of LEF-11 examined by Western blotting. BmN-SWU1 cells were co-transfected with LEF-11 and candidate protein. At 48 hours after transfection, cells were lysed and immunoprecipitation performed with α-FLAG/HA, and the bound of target protein using α-HA/FLAG to detected. The label on the top of each panel shows the antibodies used for immunoprecipitation. The labels on the right of each panel show the antibodies used for analysis of Western blotting. The apparent molecular size of each band is shown on the left of each panel.

To analyze the regulatory mechanism of LEF-11 on viral multiplication, immunoprecipitation assays were performed to identify the binding partners of LEF-11. BmN-SWU1 cells were infected with vBmlef11^cMYC^ and IP was performed using α-cMYC or mouse IgG antibody. The results showed that protein samples immunoprecipitated with α-cMYC had obvious differences in bands compared with IgG control. These proteins of 3 differential bands were located at 100 kDa, 60–70 kDa and 45–50 kDa, respectively (Fig. 1A). Protein bands were excised and subjected to digestion, and then analysis followed by tandem mass spectrometry (MS/MS). A total of 8 related proteins were screened by protein peptides and molecular mass analysis. These results showed that 5 candidate proteins with the corresponding sizes were identified in *Bombyx mori* and only 3 candidate proteins were identified from BmNPV by bioinformatics analysis. The candidate proteins include *Bombyx mori* ATAD3A, HSPD1, PP2A, Actin, PP5 and BmNPV LEF-8, LEF-3, and Chitinase protein (see Table 1 for specific information on all candidate proteins).

In order to confirm the interaction between LEF-11 and candidate proteins, we constructed candidate proteins expression plasmid containing FLAG tag sequences and LEF-11 protein expression plasmid containing HA tag sequences. The LEF-11 expression vector pIZ-LEF-11^HA^ and candidate proteins expression vectors (pIZ-LEF-8^FLAG^, pIZ-LEF-3^FLAG^, pIZ-Chitinase^FLAG^, pIZ-ATAD3A^FLAG^, pIZ-HSPD1^FLAG^, pIZ-PP2A^FLAG^, pIZ-Actin^FLAG^, and pIZ-PP5^FLAG^) were co-transfected in BmN-SWU1 cells, respectively. At 72 h.p.t., total cell lysates were lysed in IP buffer, and incubated with α-HA monoclonal antibody and control IgG in Binding buffer. Then the samples were incubated together with Dynabeads protein A + G and washed in Elution buffer. Immunoprecipitation analysis showed specific LEF-11^HA^ bands in the input lanes and α-HA, which were not seen in the IgG lanes using α-HA pull-down antibody (Fig. 1B,C). The specific bands ATAD3A^FLAG^ and HSPD1^FLAG^ were also visualized in the input lanes and α-HA pull-down antibody lanes with α-FLAG antibody. Combined, the above results suggest that LEF-11^HA^ interacted with ATAD3A^FLAG^ and HSPD1^FLAG^ (Fig. 1B,C), but not with LEF-8^FLAG^, LEF-3^FLAG^, Chitinase^FLAG^, PP2A^FLAG^, PP5^FLAG^ and Actin^FLAG^ (Fig. S1A). Meanwhile, the total cell lysates were also incubated with α-FLAG monoclonal antibody and indicated that ATAD3A^FLAG^ and HSPD1^FLAG^ interacted with LEF-11^HA^ through immunoprecipitation and Western blotting analysis (Fig. 1B,C). Further results showed that the interaction of LEF-11 with ATAD3A and HSPD1 co-localized in the nucleus using immunofluorescence assay (Fig. S1B). These results indicated that ATAD3A and HSDP1 may be important proteins involved in the regulation of LEF-11 on virus infection.

### LEF-11 induces the expression of ATAD3A and HSPD1

ATAD3A contains a highly conserved AAA modules sequence, which belongs to the AAA+ protein and ATPase family members (Fig. S2A and S2B)^28^,^29^. HSPD1 contains a highly conserved Cpn-TCP160 functional domain and plays an essential role in protein folding (Fig. S3A and S3B)^30^,^31^,^32^,^33^,^34^. In order to investigate the role of ATAD3A and HSPD1 after BmNPV infection, we first examined the expression of *BmATAD3A* and *BmHSPD1* upon BmNPV infection in *Bombyx mori* cells by qRT-PCR. Relative transcript levels indicated that the expression of *BmATAD3A* was significantly up-regulated upon infection of BmNPV (Fig. S2C and S2D). The results showed that the expression level of *BmHSPD1* upon BmNPV infection was significantly up-regulated at 12 and 24 h.p.i., and the expression levels were maintained at a stable level compared with Mock-transfection controls (Fig. S3C and S3D).

**Figure 2:**
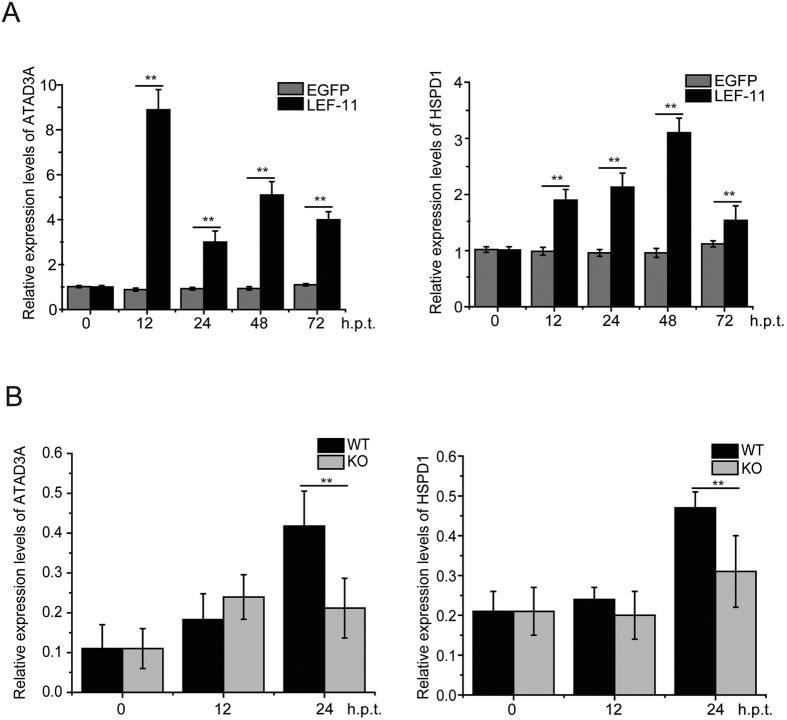
LEF-11 induces the expression of ATAD3A and HSPD1. (**A**) RT-PCR analysis of *BmATAD3A* or *BmHSPD1* transcription in LEF-11 transfected cells. At the indicated time points, cells were harvested and total RNA was prepared and reverse transcription reactions were performed with SYBR Select Master Mix Reagent. (**B**) RT-PCR analysis of *BmATAD3A* or *BmHSPD1* transcription in WT and KO transfected cells. Error bars indicate standard deviations from the mean. NS, not significant; **represents statistically significant differences at the level of *P* < 0.01.

To further illustrate the relationship between BmNPV LEF-11 with ATAD3A and HSPD1 of the *Bombyx mori* in virus multiplication, we examined the effect of LEF-11 only on the expression of *BmATAD3A* and *BmHSPD1* gene. BmN-SWU1 cells were transfected with *lef-11* gene expression plasmids. At various time–points following transfection, cells were harvested and total RNA was prepared and subjected to qRT-PCR analysis with the indicated primers. At 0 h.p.t., a similar amount of *BmATAD3A* or *BmHSPD1* transcription was detected in cells transfected with *lef-11* gene expression plasmids and control cells transfected with pIZ-V5/His plasmids cells. As shown in Fig. 2A *BmATAD3A* or *BmHSPD1* transcription levels significantly increased in *lef-11* gene expression plasmids transfected cells. *BmATAD3A* transcription level was 9.2, 3.3, 4.5 and 4.1-fold greater in LEF-11 overexpressed cells than control pIZ-V5/His transfected cells at 12, 24, 48 and 72 h.p.t., respectively (Fig. 2A). In addition, *BmHSPD1* transcription level increased 1.9, 2.3, 3.2 and 1.6-fold in *lef-11* gene expression plasmids transfected cells compared with control pIZ-V5/His transfected cells at 12, 24, 48 and 72 h.p.t., respectively (Fig. 2A). These results indicated that BmNPV *lef-11* gene expression could induce the levels of transcription of *BmATAD3A* and *BmHSPD1*.

To clarify whether the transcription levels of *BmATAD3A* or *BmHSPD1* were indeed activated by the BmNPV *lef-11* gene expression, cells transfected with wild type bacmid (WT) or *lef-11* gene knockout bacmid (KO) were harvested to assess target genes transcription levels at different time-points. As shown in Fig. 2B *BmATAD3A* or *BmHSPD1* transcription levels significantly increased at 24 h.p.t. compared with 0 h.p.t. of WT-transfected cells (Fig. 2B). In *lef-11*-KO cells at 24h.p.t., *BmATAD3A* transcription levels were not significantly increased compared with 0 h.p.t. Though *BmHSPD1* transcription levels slowly increased, there was no remarkable difference compared to WT-transfected cells (Fig. 2B). These results suggested that BmNPV LEF-11 indeed significantly affected the transcription of *BmATAD3A* and *BmHSPD1* in BmN-SWU1, which implies that BmNPV LEF-11 has the ability to induce the expression of host ATAD3A and HSPD1.

### ATAD3A and HSPD1 contribute to virus multiplication

**Figure 3:**
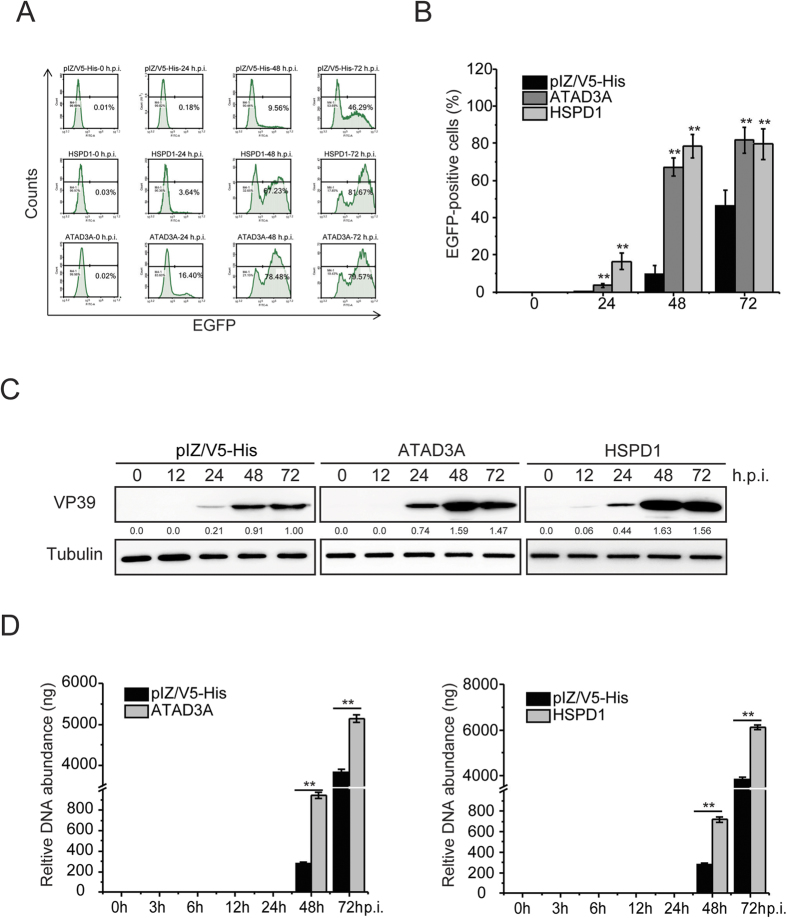
ATAD3A and HSPD1 contribute to virus multiplication. (**A**) BmNPV multiplication was measured on ATAD3A and HSPD1 overexpression cells by flow cytometric analysis and ATAD3A, HSPD1 and Mock were infected with BmNPV at different time-points. (**B**) Statistical analysis of the EGFP+ positive cells in the indicated stable cell lines after BmNPV infected at different time-points. **Represents statistically significant differences at the level of *P* < 0.01. (**C**) Western blotting analysis of VP39 protein synthesis after overexpression with ATAD3A, HSPD1 and Mock at different times in BmNPV-infected BmN-SWU1 cells. (**D**) BmN-SWU1 cells were treated with indicated plasmid and infected with BmNPV at MOI of 10. At 0, 3, 6, 12, 24, 48 and 72 h.p.i., total intracellular DNA was extracted from infected cells. BmNPV copy numbers were determined by qPCR. Each data point was determined from the mean of three independent replicates. NS, not significant. **Represents statistically significant differences at the level of *P* < 0.01.

To identify whether ATAD3A and HSPD1 were involved in BmNPV production, ATAD3A and HSPD1 stable expression cell lines were generated via supplementation with 400 μg/ml Zeocin TC100 medium (Fig. S4). Then the relative levels of BmNPV (without EGFP) in pIZ-V5/His, ATAD3A and HSPD1 stables cell lines were examined, respectively. After infection with BmNPV in the indicated cells lines, the virus multiplication of BmNPV was examined using a flow cytometry analysis at 0, 24, 48 and 72 h.p.i., respectively. As shown in Fig. 3A, stable cell lines of ATAD3A and HSPD1 significantly increased BmNPV multiplication compared with pIZ-V5/His cell lines at different time points post infection (Fig. 3A). Statistical analysis results also showed that the average number of EGFP+ positive cells increased significantly after BmNPV infection with stable cell lines of ATAD3A and HSPD1 during specific periods of time (Fig. 3B). Western blot analyses showed that BmNPV VP39 protein increased after infection with stable cell lines of ATAD3A and HSPD1 at different time-points (Fig. 3C). Compared to the pIZ-V5/His cell lines, the stable cell lines of ATAD3A and HSPD1 expression VP39 increased at 24 h.p.i. and continued until 72 h.p.i. (Fig. 3C).

Furthermore, to examine whether ATAD3A and HSPD1 were involved in BmNPV infection, we analyzed the changes of BmNPV DNA replication. BmNPV DNA replication was detected to determine the effect of target genes on virus proliferation. At 0 h.p.i., a similar amount of viral DNA was detected in the stable cell lines of ATAD3A, HSPD1 and pIZ-V5/His, which indicated that the amount of transfection bacmid in all of cell lines was similar. The viral DNA replication levels increased significantly at different time-points. The amount of the viral DNA accumulated in stable cell lines of ATAD3A was increased by 335.2% and 133.8% compared with pIZ-V5/His cell lines at 48 and 72 h.p.i., respectively (Fig. 3D). The amount of the viral DNA accumulated in stable cell lines of HSPD1 cells was increased by 341.0% and 150.2% compared with BmN-SWU1 cell lines at 48 and 72 h.p.i., respectively (Fig. 3D). Together, these results suggest that ATAD3A and HSPD1 have the ability to promote BmNPV DNA accumulation in viral infection cells.

### ATAD3A and HSPD1 are required for virus multiplication

**Figure 4:**
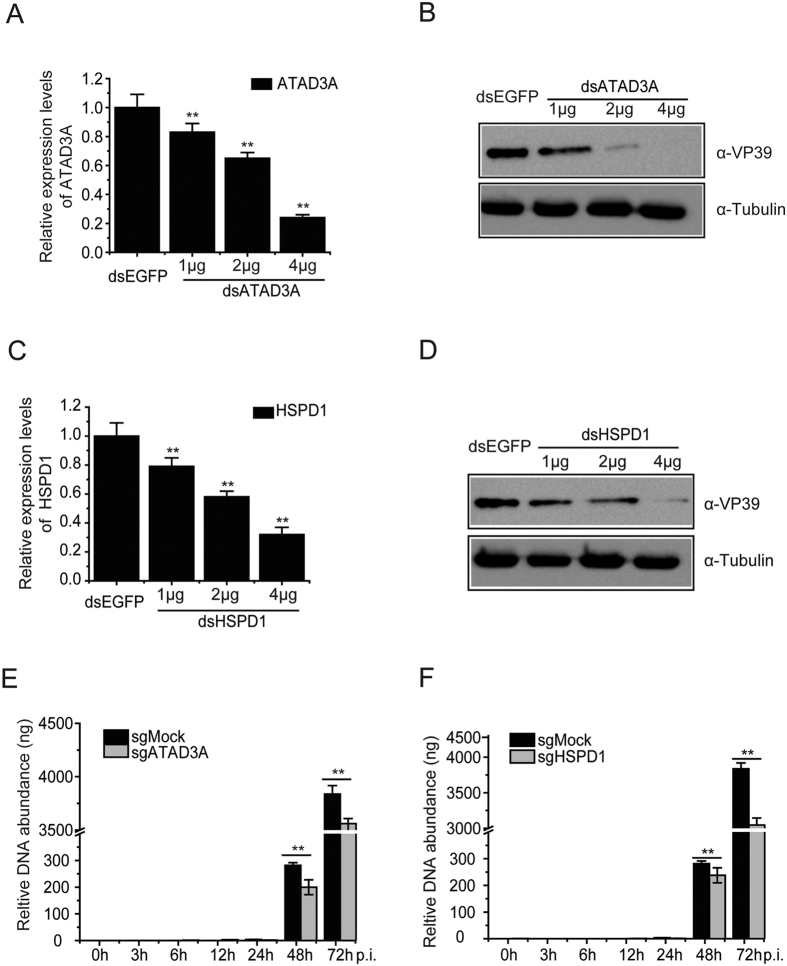
ATAD3A and HSPD1 are required for virus multiplication. (**A**) Knockdown of ATAD3A in BmN-SWU1 cells was confirmed by RT-PCR. BmN-SWU1 cells were transfected with dsRNA for dsEGFP (2 μg), dsATAD3A (1 μg), dsATAD3A (2 μg) and dsATAD3A (4 μg). After 48 h.p.t., the transcript levels of ATAD3A were examined by RT-PCR. (**B**) Effect of dsATAD3A mediated knockdown of *BmATAD3A* on BmNPV VP39 protein expression. After 48 h.p.t., BmN-SWU1 cells were infected with BmNPV at MOI of 1, and VP39 and Tubulin expression levels was assessed. (**C**) Effect of dsHSPD1 mediated knock down of *BmHSPD1* on HSPD1 transcription level. BmN-SWU1 cells were transfected with dsRNA for dsEGFP (2 μg), dsHSDP1 (1 μg), dsHSPD1 (2 μg) and dsHSDP1 (4 μg). After 48 h.p.t., the transcript levels of HSPD1 were examined by RT-PCR. (**D**) After 48 h.p.t., the expression levels of VP39 were examined by Western blotting. Tubulin expression levels as control was assessed. (**E**) Effects of viral DNA replication of knockout ATAD3A on CRISPR/Cas9 system. Transfection with CRISPR/Cas9 system and infected with BmNPV at MOI of 1. (**F**) Effects of viral DNA replication of knockout HSPD1 on CRISPR/Cas9 system. Transfection with CRISPR/Cas9 system and infected with BmNPV at MOI of 1. At different time-points, total DNA was isolated from knockout cell and quantified by Q-PCR. **P < 0.01.

To determine whether ATAD3A was important for virus multiplication, we performed knockdown of ATAD3A and HSPD1 in *Bombyx mori* cells, and analyzed the changes of viral protein expression. BmN-SWU1 cells were transfected with dsATAD3A, dsHSPD1 or dsEGFP for 48 h and we analyzed the changes in *BmHSPD1* and *BmATAD3A* transcription level. A significant reduction was observed in the relative expression level of ATAD3A and HSPD1 with the increase in the amount of dsATAD3A and dsHSPD1 compared with the control dsEGFP (Fig. 4A,C). Additionally, there ATAD3A and HSPD1 knockdown did not affect cell cycle progression by FACS assay (Fig. S5). Western blot analyses showed that the knockdown of ATAD3A and HSPD1 resulted in reduction of BmNPV VP39 protein expression levels concurrent with the increase in the expression of dsATAD3A and dsHSPD1 (Fig. 4B,D). In contrast, the expression levels of Tubulin were not affected by dsRNA transfection. These results suggested that ATAD3A and HSPD1 were indeed involved in BmNPV protein production and were required for virus multiplication.

To further verify the effects of ATAD3A and HSPD1 protein on BmNPV infection, ATAD3A and HSPD1 were deleted through CRISPR/Cas9 gene editing system. Knockout ATAD3A and HSPD1 were confirmed through analyzing sequence by TA-clone (Fig. S6). The amount of viral DNA replication was reduced at 24 h.p.i. after knockout of ATAD3A and HSPD1 in comparison with control sgMock (Fig. 4E,F). Based on the results thus far, we conclude that the ATAD3A and HSPD1 were required for virus efficient multiplication.

### ATAD3A interacts with HSPD1

**Figure 5:**
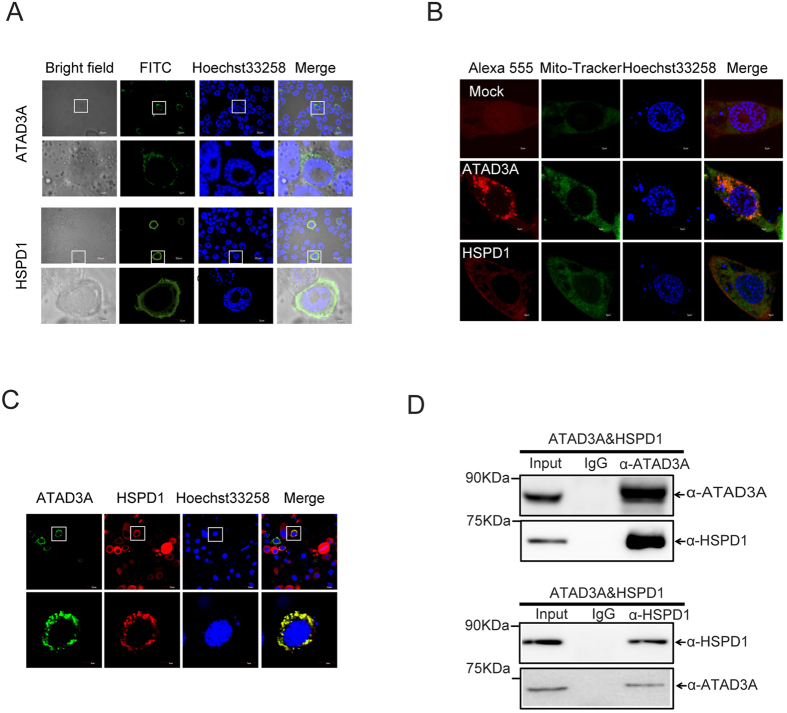
ATAD3A interacts with HSPD1. (**A**) Subcellular localization of ATAD3A and HSPD in BmN-SWU1 cells. ATAD3A and HSPD1 were stained with FITC-labeled and Hoechst33258 at 48 h post-transfection in BmN-SWU1 cells. Green fluorescence represents fluorescent represent ATAD3A and HSPD1, blue fluorescence represents the nucleus. Scale bar: 5 μm. (**B**) Mitochondria co-location of ATAD3A and HSPD1 in BmN-SWU1 cells. ATAD3A and HSPD1 were stained with Alexa 555-labeled, mitochondria-tracker and Hoechst33258 at 48 h post-transfection in the BmN-SWU1 cells. Red fluorescence represents ATAD3A and HSPD1, Green fluorescence represents Mito-Tracker, and blue fluorescence represents the nucleus. Scale bar: 5 μm. (**C**) Co-localization of ATAD3A and HSPD1 in BmN-SWU1 cells. ATAD3A and HSPD1 stained with Alexa 555-labeled anti-HSPD1, FITC-labeled anti-ATAD3A and Hoechst33258 at 48 h post-transfection in the BmN-SWU1 cells. Red fluorescence represents HSPD1, Green fluorescence represents ATAD3A, and blue fluorescence represents the nucleus. Scale bar: 5 μm. (**D**) Co-immunoprecipitation of ATAD3A and HSPD1 examined by Western blotting. The label on the top of each panel shows the antibodies used for immunoprecipitation. The labels on the right of each panel shows the antibodies used for analysis of Western blotting. The apparent molecular size of each band is shown on the left of each panel.

To further determine the function of ATAD3A and HSPD1 in silkworm cells, we used confocal microscopy to analyze their localization after transfection in BmN-SWU1 cells. Immunofluorescence assay showed that ATAD3A and HSPD1 were mainly distributed in the cytoplasm (Fig. 5A). Mitochondria co-localization assay showed that they co-localize with mitochondria as assessed by Mito-Tracker Green (Fig. 5B). In contrast, the control DsRed protein fluorescence signals were observed in the cell nuclei and cytoplasm. These results further suggest that the endogenous ATAD3A and HSPD1 are mitochondrial proteins in silkworm cells.

Previous studies showed that ATP-dependent proteases combine chaperone-like and proteolytic activities, and HSPD1 is essential for the folding and assembly of the target protein in the presence of ATP^33^,^34^,^35^. In order to confirm the relationship of both ATAD3A and HSDP1 in the *Bombyx mori*, BmN-SWU1 cells were fixed in coverslips glasses, and co-stained with monoclonal antibodies against ATAD3A and HSPD1. Immunofluorescence assay shows that the endogenous ATAD3A and HSPD1 were co-localized in the cytoplasm (Fig. 5C). Further, the total cell lysates were collected in Western and IP buffer, and incubated with α-ATAD3A monoclonal antibody and control IgG in Binding buffer. Then the samples were incubated together with Dynabeads protein A + G and washed in Elution buffer. Immunoprecipitation analysis showed specific ATAD3A bands appeared in the input lanes and α-ATAD3A, which were not seen in the IgG lines using α-ATAD3A pull-down antibody (Fig. 5C). The specific HSPD1 bands were also obtained in the input lanes and α-ATAD3A pull-down antibody lanes with α-HSPD1 antibody. The above results show that ATAD3A could directly interact with HSPD1 in *Bombyx mori* cells (Fig. 5D). Combined with previous research results of their function as mitochondrial proteins, our data suggests that HSPD1 might be involved in the regulation of ATAD3A ATPase by directly binding to ATAD3A or as a molecular chaperone of ATAD3A.

### ATAD3A is involved in the synthesis of HSPD1

**Figure 6:**
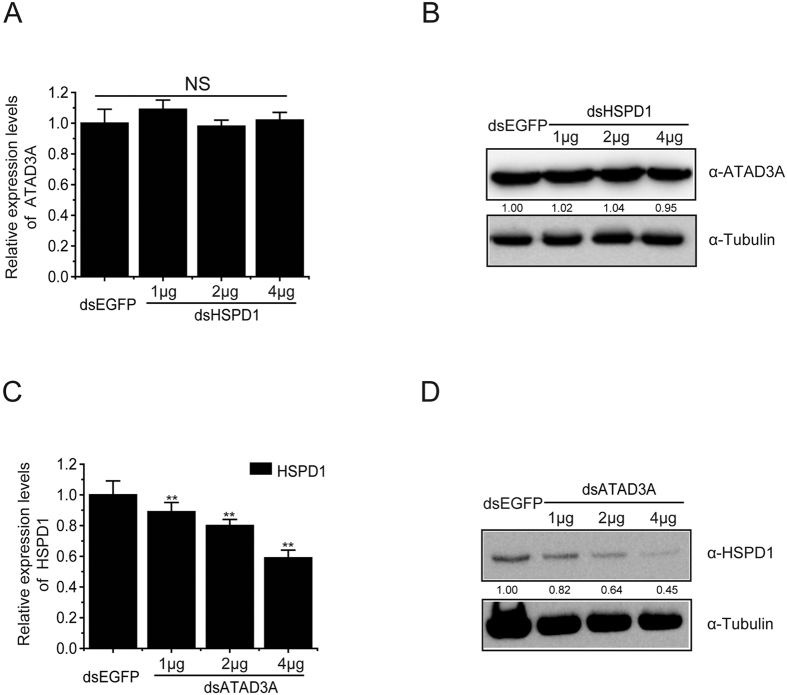
ATAD3A mediates HSPD1 protein stability. (**A**) Effect of dsATAD3A mediated knockdown of *BmATAD3A* on HSPD1 transcription level. BmN-SWU1 cells were transfected with dsRNA for dsEGFP (2 μg), dsATAD3A (1 μg), dsATAD3A (2 μg) and dsATAD3A (4 μg). After 48 h.p.t., the transcript levels of HSPD1 were examined by RT-PCR. (**B**) After 48 h.p.t., the expression levels of HSPD1 were examined by Western blotting. Tubulin expression levels as control was assessed. (**C**) Effect of dsHSPD1 mediated knockdown of *BmHSPD1* on ATAD3A transcription level. BmN-SWU1 cells were transfected with dsRNA for dsEGFP (2 μg), dsHSPD1 (1 μg), dsHSPD1 (2 μg) and dsHSPD1 (4 μg). After 48 h.p.t., the transcript levels of ATAD3A were examined by RT-PCR. (**D**) After 48 h.p.t., the expression levels of ATAD3A were examined by Western blotting. Tubulin expression levels as control was assessed.

To determine whether ATAD3A had a role in HSPD1 function, we used RT-PCR and Western blotting to analyze the knockdown of the ATAD3A and HSPD1 in BmN-SWU1 cells, respectively. Results showed that ATAD3A transcription levels and protein levels had no obvious changes after silencing HSPD1 with the dsHSPD1 compared with the control dsEGFP (Fig. 6A,B). These results indicated that HSPD1 has no significant effect on the expression of ATAD3A protein. In order to further confirm the regulatory relationship between ATAD3A and HSPD1, HSPD1 transcription levels and protein levels were detected in dsATAD3A transfected cells. Results showed that HSPD1 transcription levels and protein levels were reduced in dsATAD3A transfected cells compared with the control dsEGFP transfected cells (Fig. 6C,D). These results indicated that ATAD3A was important for the expression of HSPD1 protein. Combined with the results of their co-localization, we speculated that ATAD3A is an important regulator of HSPD1 protein expression and ATAD3A may be an upstream regulatory protein of HSPD1.

## Discussion

In the last decade, viral host interaction studies have shown that many viruses have evolved to alter host cell pathways and processes to ensure optimal environments for their multiplication and replication^1^,^17^. A common feature of many viruses is in their capability to induce large scale alteration in host cellular energy metabolism for their replication, and as such, some virus proteins that are capable of this function has been identified^4^,^15^. However, to date, the regulation of energy metabolism induced by baculovirus infection is still largely unknown^13^,^17^. In this study, our results demonstrate for the first time, a model that proposes the role of baculoviruses LEF-11 in the induction of ATAD3A and HSPD1 to promote virus multiplication in silkworm cells. To the best of our knowledge, this is the first study that provides direct evidence that BmNPV induces ATPase family members ATAD3A and HSPD1 of host for their replication in infection cells.

A common feature of viruses is that they can exploit the host apparatus for their multiplication in infected cells. For example, previous studies have reported that baculovirus PK2 could subvert insect eF2α kinase function by engaging the N-lobe domain, and the structural protein ODV-EC27 can simulate the Cyclin B like function by interacting with CDC2 and CDK6 result in arresting of the host cell cycle at G2/M phase^12^,^36^. Nonetheless, how viruses hijack various pathways of energy metabolism are still largely unknown. Herein, we demonstrated that baculovirus LEF-11 could directly interact with *Bombyx mori* ATAD3A and control the host cells for production of new viruses in infected cells (Figs 1 and 3). These results provide further validation that a large number of processes require the host factor support of host cells during virus infection^9^,^13^,^15^. Combined with these findings that the expression of ATAD3A and its molecular chaperone HSPD1 can be activated by LEF-11, and LEF11-KO bacmid could not activate the expression of these 2 genes, our results further confirm that baculovirus LEF-11 has the ability to hijack ATAD3A and its molecular chaperone HSPD1 in host cells for their multiplication (Fig. 2A,B). However, the specific mechanism that baculovirus uses hijacking the ATPase requires further study.

ATAD3A belongs to ATPase family of mitochondrial membrane proteins which is involved in mitochondrial fragmentation, cellular autophagy and ER-mitochondrial communication^28^,^29^,^37^. In this study, results demonstrated that ATAD3A expression was closely associated with BmNPV infection similar with a previously reported study on HPV infection (Fig. S2C)^38^. Interestingly, additional results demonstrated that overexpression of ATAD3A promotes virus multiplication in the *Bombyx mori* cells (Fig. 3). These results further explain why this protein expression correlates with virus infection and cancer in the mammalian cells^38^,^39^. According to the phenomenon that propagation and replication of baculovirus would be inhibited through integrating silencing ATAD3A with the experimental results mentioned above, we speculate that virus could use ATAD3A to utilize host factor energy for their replication (Fig. 4). The ATPase activity of molecular chaperones HSPD1 plays an important role in the folding of the newly synthesized proteins and ATP hydrolysis^33^,^40^. Furthermore, the molecular chaperones HSPD1 of ATAD3A also have the ability to promote virus multiplication, which further affirms the speculation mentioned above that baculovirus could induce ATAD3A to promote virus multiplication.

A supposition in which baculovirus LEF-11 induces cellular factor, including ATAD3A and HSPD1, to promote virus replication and multiplication is consistent with our results. Following viral entry of host cells, immediate early gene expression and viral initiation DNA replication occurs^23^,^41^. After initiating DNA replication of baculovirus, the LEF-11 protein was expressed and hijack host ATPase family members ATAD3A and HSPD1^39^. Then ATAD3A could directly provide ATPase and the folding activity of chaperone protein HSPD1 correlating with ATP hydrolysis activity (Fig. 5)^33^,^34^. Our previous study showed that LEF-11 mainly localized in the nucleus, ATAD3A and HSPD1 proteins mainly localized in the cytoplasm by immunofluorescence analysis. However, LEF-11 could co-localization with ATAD3A and HSPD1 proteins in the nucleus after their co-expression. Therefore, we speculate that baculovirus LEF-11 may hijack ATAD3A and HSPD1 proteins into the nucleus to regulate viral DNA replication^27^. Furthermore, we hypothesized that baculovirus LEF-11 could induce both of them to form a ternary complex in baculovirus infection, to provide ATP for the virus. Finally, baculovirus hijacks ATP of host cells to promote virus multiplication. In the present study, our research revealed that knockdown of ATAD3A, HSPD1 and LEF-11 significantly reduced the protein expression and DNA replication of the virus (Fig. 4)^23^. This indicated that the ATAD3A and HSPD1 are required for the efficient multiplication of BmNPV.

In summary, our study has shown that baculovirus LEF-11 hijacks the host ATAD3A and its molecular chaperone HSPD1 to induce energy metabolism, which is required for virus multiplication. Additionally, this study, for the first time, defines a baculovirus protein that induces host ATPase to promote virus multiplication in silkworm cells. Our ongoing research is currently exploring the transport and the regulatory mechanisms of host ATP that are hijacked by the virus following BmNPV infection in the host cells. By identifying how viruses induce host cellular protein synthesis and signaling pathway for their replication, we may be able to further analyze the mechanism of baculovirus and host interaction.

## Materials and Methods

### Cells culture and transient transfections

*Bombyx mori* ovary cell line BmN-SWU1 was established and preserved by our laboratory, and grown in TC-100 medium (United States Biological, USA) supplemented with 10% fetal bovine serum (FBS) (Gibco, USA), 100 U/ml penicillin and 100 μg/ml streptomycin^42^. After the cells were grown to 80% confluency, the plasmids were transfected with the cells using the X-tremeGENE HP DNA Transfection Reagent (Roche, Switzerland). Briefly, 1 × 10^5^ cells were transfection with 0.8 μg of ATAD3A, HSPD1 and LEF-11 expression plasmids and at 6–8 hour post transfection, the medium was replaced with medium containing 10% FBS.

### Virus and infection

The wild-type (WT) recombinant virus in which the *polh* locus was replaced with A4^prm^-EGFP-POLY (BmNPV-EGFP) was constructed as described previously^23^. The *lef-11* knockout (KO) bacmid and repair bacmid (vBmlef11^cMYC^) was previously created in our laboratory^23^. BmNPV WT isolate T3 and BmNPV-EGFP were cultured in BmN-SWU1 cells, and viral titers were determined by 50% tissue culture infective doses (TCID_50_) assay. The BmN-SWU1 cells or transfected cells were incubated at 27 °C and infected with viruses at a multiplicity of infection (MOI) of 1. The time of infection was defined as zero when the virus was inoculum 1 hour was replaced with fresh TC100 medium.

### Antibodies

Affinity-purified polyclonal antibodies to VP39 using protein A column were raised in rabbit and maintained in our lab. The commercial primary antibodies of anti-FLAG (1:5000; Sigma, USA), anti-HA (1:5000; Sigma, USA), anti-Tubulin (1:5000; Sigma, USA Sigma), anti-HSPD1 (1:2000; Abcam, UK), anti-ATAD3A (1:2000; Abcam, UK) and anti-EGFP (1:2000; Abcam, UK) were used in this study. The commercial secondary antibodies used were HRP-conjugated goat anti-rabbit IgG (BIO-RAD, USA) and HRP-conjugated goat anti-mouse IgG (BIO-RAD, USA). Immunofluorescence fluorescent secondary antibodies were Alexa 555-conjugated goat anti-mouse IgG or FITC-conjugated goat anti-rabbit IgG (1:500; Life Technologies, USA) and Hoechst 33258 (1:500; Life Technologies, USA).

### Plasmid construction

The expression plasmids pIZ-ATAD3A^FLAG/HA^, pIZ-HSPD1^FLAG/HA^, and pIZ-LEF-11^FLAG/HA^ were constructed for this study. Briefly, to generate plasmid pIZ-ATAD3A^FLAG/HA^, pIZ-HSPD1^FLAG/HA^, and pIZ-LEF-11^FLAG/HA^ which encodes target gene with a C-terminal and N-terminal FLAG or HA epitope, the target sequence of plasmid was PCR-amplified and inserted into the opIE2 promoter-based vector pIZ-V5/His (Invitrogen, USA). All target sequences were inserted at corresponding restriction enzyme site and verified by sequencing the plasmids. All primers used in this study are presented in the S1 Table.

### Co-immunoprecipitation (Co-IP) assay

Total cell lysates in 1 ml of Western and IP buffer [20 mM Tris(pH7.5), 150 mM NaCl, 1% Triton X-100] (Beyotime, China), were incubated with α-FLAG/α-HA monoclonal antibody and control IgG in Bing buffer (Life technologies, USA) for 30 minutes at 4 °C. The samples were incubated together with Dynabeads protein A + G (Life technologies, USA) for 30 minutes of rotation at 4 °C.Then the proteins were collected by centrifugation (13,400 × g) and washed with Elution buffer (Life technologies, USA). After 3 times repeated centrifugation and washed, transferred protein sample to a new Eppendorf tube. SDS-PAGE and Western blotting analysis were performed.

### LC-MS/MS analysis

After the protein sample was loaded on SDS-PAGE gel and separated, the gels were stained with silver staining. Then the protein samples immunoprecipitated with α-cMYC had obvious differences in bands compared with IgG control. These proteins of 3 differential bands were located on at 100 kDa, 60–70 kDa and 45–50 kDa, respectively. Protein samples were excised and subjected to trypsin digestion, and then analysis followed by tandem mass spectrometry (MS/MS). Then, the peptides were eluted onto a 10 cm analytical C18 column (inner diameter 75 μm) packed in-house. The peptides were subjected to Nano electrospray ionization followed by tandem mass spectrometry (MS/MS) in a Q EXACTIVE (Thermos Fisher Scientific, San Jose, CA) coupled online to the HPLC. Peptides were selected for MS/MS using high-energy collision dissociation (HCD) operating mode with a normalized collision energy setting of 27.0; ion fragments were detected in the Orbit rap at a resolution of 17500.

### Western Blot analysis

The BmN-SWU1 cells (2 × 10^5^) were plated in 24-well plates, and transfected with 0.8 μg plasmid. At the indicated time points, cells were harvested for Western blotting. The cell samples were lysed using Western and IP buffer [20mM Tris(pH7.5), 150 mM NaCl, 1% Triton X-100] (Beyotime, China). The total protein concentration was determined using a BCA Protein Assay Kit (Beyotime, China). After SDS-PAGE, the proteins were transferred onto a nitrocellulose membrane (Roche, Switzerland) and incubated with indicated primary antibodies, respectively. Then, the membrane was further incubated with HRP-labeled secondary antibodies. The blots were visualized using a Clarity Western ECL Substrate (Bio-Rad, USA).

### Reverse transcription-qPCR (RT-qPCR)

BmN-SWU1 cells were plated in 6-well plates at a density of 1 × 10^6^ cells per well. After 24 hours, cells were infected with BmNPV at an MOI of 1. At the indicated time points, cells were harvested and total RNA was prepared using TRIzol reagent (Invitrogen, USA) as described previously^43^. Samples were normalized to total RNA and reverse transcription reactions were performed with SYBR Select Master Mix Reagent (Bio-Rad, USA) using specific primers (S1 Table). The *Bombyx mori sw22934* gene was used as an endogenous control. The sample analysis was performed in triplicate on the CFX96 Real-Time System.

### Screening stable cell lines

The ATAD3A, HSPD1 and pIZ-V5/His expression plasmids were transfected into BmN-SWU1cells, and then cultured with 400 μg/mL Zeocin of TC100 medium. After the stable expression of their protein, the cells were further cultured for an additional two months, which resulted in the increased expression of the target protein in the cells. Culture continued until more than 95% of the cells expressed target protein, and the obtained cell lines were used for antiviral research.

### Flow cytometry analysis

After infection with BmNPV, the cells were collected with PBS. The numbers in the right in the flow charts indicated the percentage of EGFP positive cells based on fluorescence. Then, harvested cells were analyzed for the percentage of EGFP cells using an ACEA NovoCyte flow cytometer (ACEA Biosciences, USA). The data were analyzed using Novo Express software.

### Quantitative real-time PCR (qPCR) DNA replication assay

BmN-SWU1 cells were infected with BmNPV at an MOI of 1, and at the indicated time points, the cells were washed with PBS and harvested. The total DNA samples were extracted using a Wizard Genomic DNA extraction kit (Promega, USA) according to the manufacturer’s protocol. The *gp41* copy number of BmNPV was set for analysis of viral DNA replication. The quantitative PCR was performed as previously described with SYBR Select Master Mix Reagent (Bio-Rad, USA) using *gp41* primers (S1 Table).

### Double-stranded RNA (dsRNA) synthesis

DsRNA for *BmATAD3A, BmHSPD1* and *EGFP* was generated by using a T7 RiboMAX™ Express Large Scale RNA Production System kit (Promega, USA). Primers used in this study are listed in S1 Table. BmN-SWU1 cells were plated onto six-well plates at 1 μg, 2 μg and 4 μg of 1 × 10^6^ cells per well, and immediately transfected using Lipofectamine RNAiMAX (Life technologies, USA). After 96 hours, transfected cells were infected with BmNPV at an MOI of 10. At 48 hours after infection, cells were harvested, and gene/protein expression was determined by qRT-PCR/Western blotting.

### sgRNA design and knock out analysis

The CRISPR/Cas9 system target sequence of*ATAD3A* and *HSPD1* were designed as previously described. In brief, the predicted target sequence of *ATAD3A* and *HSPD1* were analyzed online (http://crispr.dbcls.jp/) and fitted with the rules of GN19NGG sequence. Oligoes to create sgRNAs were cloned into the pSL1180-IE1-Cas9-Ser-PA-U6-sgRNA construct as previously described. Then, the BmN-SWU1 cells (2 × 10^6^) were plated in 6-well plates, and transfected with 0.8 μg sgATAD3A, sgHSPD1 and sgMock plasmid. At the indicated time points, cells were harvested for DNA replication assay. After knocking out all target genes, all the sequences of target genes were cloned into pMD-T 19 vectors, and sequenced by M13 primers (S1 Table).

### Immunofluorescence

BmN-SWU1 cells grown on coverslips (Fisher Scientific, USA) were fixed in 4% paraformaldehyde and permeabilized with 0.1% Triton X-100, washed with PBST, and blocked with 3% BSA and 10% sheep serum in PBS and then immunofluorescence assay was performed as previously describe above^27^. The cells were stained with a monoclonal α-ATAD3A or α-HSPD1 antibody and stained with Alexa 555-conjugated goat anti-mouse IgG, FITC-conjugated goat anti-mouse IgG, Hoechst 33258 and Mito-Tracker Green (Beyotime, China) as described above^43^.

### Statistical analysis

Statistical analysis was performed using GraphPad Prism5 statistical software. Statistical significance was set at *P* < 0.01. All results presented as mean ± s.d. from at least three independent experiments.

## Additional Information

**How to cite this article**: Dong, Z.-Q. *et al*. Baculovirus LEF-11 Hijack Host ATPase ATAD3A to Promote Virus Multiplication in *Bombyx mori* cells. *Sci. Rep.* **7**, 46187; doi: 10.1038/srep46187 (2017).

**Publisher's note:** Springer Nature remains neutral with regard to jurisdictional claims in published maps and institutional affiliations.

## Supplementary Material

Supplementary Dataset1

## Figures and Tables

**Table 1:** LC-MS/MS analysis of the Co-immunoprecipitation of *Bombyx mori* LEF-11.

Protein ID	Description	Size (kDa)	Protein Score	Functions
Protein Candidates Interacting with LEF-11 by LC-ESI-MS/MS				
Proteins from BmNPV				
gi|548578106|gb|AGX01266.1|	LEF-8	102.8	1183.49	The primary components of the BmNPV-encoded RNA polymerase
gi|3745945|gb|AAC63792.1|	CHITINASE	62	518.2	Chitinase serves as a molecular chaperone of pro-V-Cath, the precursor of V-Cath, for its proper folding and transportation in the ER. Chitinase has an influence on the polyhedra releasing and cell lysis
gi|3745893|gb|AAC63740.1|	LEF-3	45.2	768.71	Viral DNA replication and facilitates P143 transport into the nucleus
Proteins from *Bombyx mori*				
BGIBMGA007349-PA	HSPD1 (60 kDa heat shock protein mitochondrial)	61.2	151.17	Implicated in mitochondrial protein import and macromolecular assembly
BGIBMGA000542-PA	ATAD3A (ATPase family AAA domain-containing protein 3A)	70.7	55.36	Essential for mitochondrial network organization, mitochondrial metabolism and cell growth at organism and cellular level
BGIBMGA002237-PA	PP2A (Serine/threonine- protein phosphatase)	66.1	50.73	Protein phosphatase 2 (PP2), targeting oncogenic signaling cascades, such as Raf, MEK, and AKT.
BGIBMGA004807-PA	PP5 (Serine/threonine- protein phosphatase 5)	56.7	32.22	Ser/thr protein phosphatase 5 (PP5) inactivates hypoxia-induced activation of an ASK-1/MKK-4/JNK-signaling cascade.
BGIBMGA013945-PA	Actin, muscle-type A1	42.1	584.62	Actins are highly conserved proteins that are involved in various types of cell motility and are ubiquitously expressed in all eukaryotic cells.

LC-MS/MS analysis of the co-immunoprecipitation of BmNPV LEF-11.
