# Supplementary material for: Baculovirus LEF-11 Hijack Host ATPase ATAD3A to Promote Virus Multiplication in Bombyx mori cells
Source: Sci Rep. 2017 Apr 10;7:46187. doi: 10.1038/srep46187 (PMC5385504; doi:10.1038/srep46187)
Supplement: Supplementary Dataset1 [file srep46187-s1.docx]

**Baculovirus LEF-11 Hijack Host ATPase ATAD3A to Promote Virus Multiplication in *Bombyx mori***

Zhan-Qi Dong^a†^, Nan Hu^a†^, Fei-Fan Dong^a†^, Ting-Ting Chen^a^, Ya-Ming Jiang^a^, Peng Chen^a^, Cheng Lu^a,b#^, Min-Hui Pan^a,b#^

^a^State Key Laboratory of Silkworm Genome Biology, Southwest University, Chongqing 400716, China^a^;

^b^Key Laboratory for Sericulture Functional Genomics and Biotechnology of Agricultural Ministry, Southwest University, Chongqing 400716, China^b^;


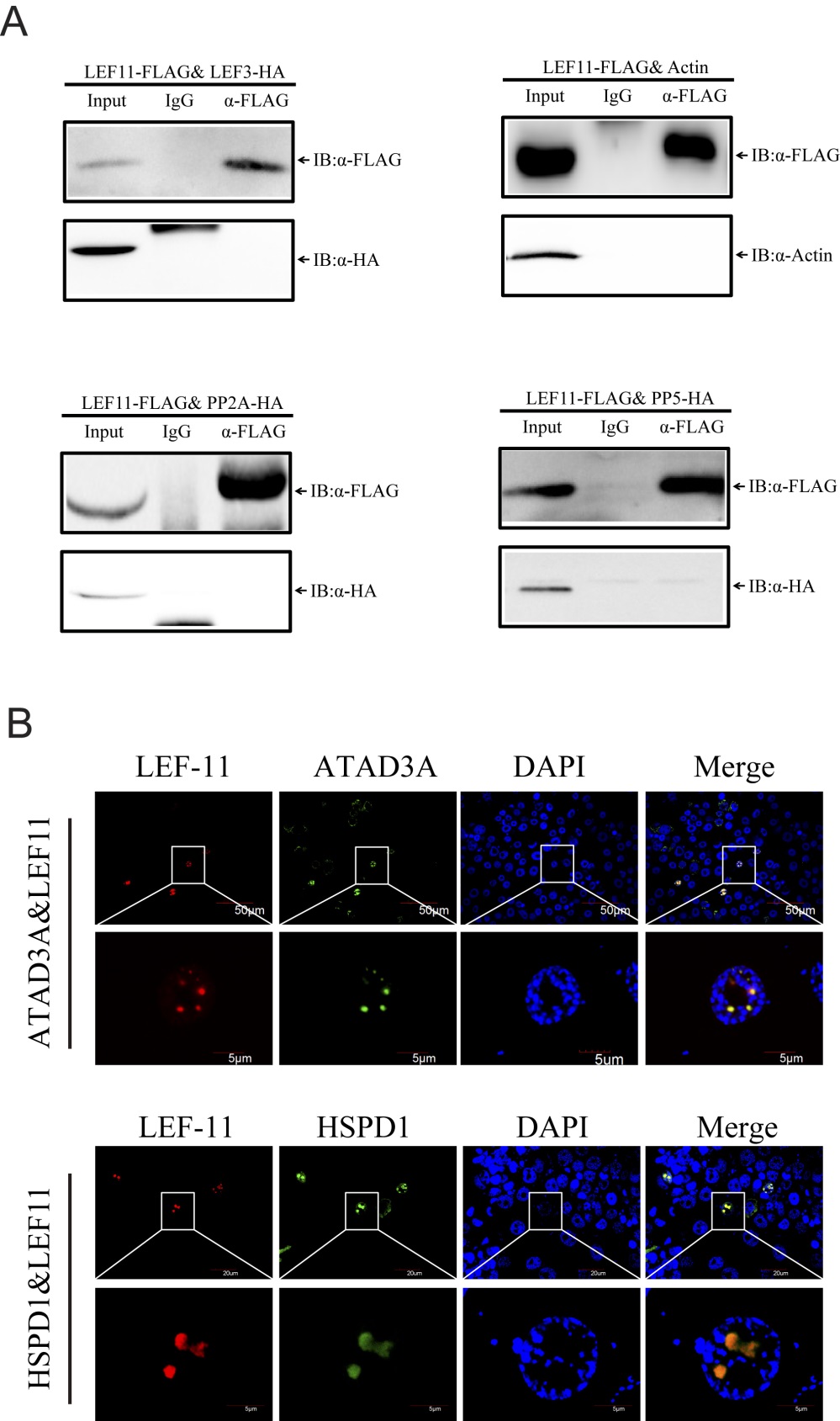


**S1 Fig. Identification of LEF-11 interaction proteins.** (A) Co-immunoprecipitation of LEF-11 tested by Western blotting. BmN-SWU1 cells were co-transfected with LEF-11 and candidate protein. At 48 after transfection, cells were lysed and immunoprecipitated with ɑ-FLAG/HA, and the bound target protein was detected using ɑ- HA/FLAG. The label on the top of each panel shows the antibodies used for immunoprecipitation. The labels on the right of each panel shows the antibodies used for Western blot analysis. (B) Co-localization of LEF-11with ATAD3A and HSPD1 in BmN-SWU1 cells. ATAD3A and HSPD1 were stained with Alexa 488-labeled anti-HA and anti-HA, LEF-11 stained with Alexa 555-labeled anti-Flag and Hoechst33258 at 48 h post-transfection in the BmN-SWU1 cells. Red fluorescence represents LEF-11, Green fluorescence represents ATAD3A and HSPD1, and blue fluorescence represents the nucleus. Scale bar: 5 μm.


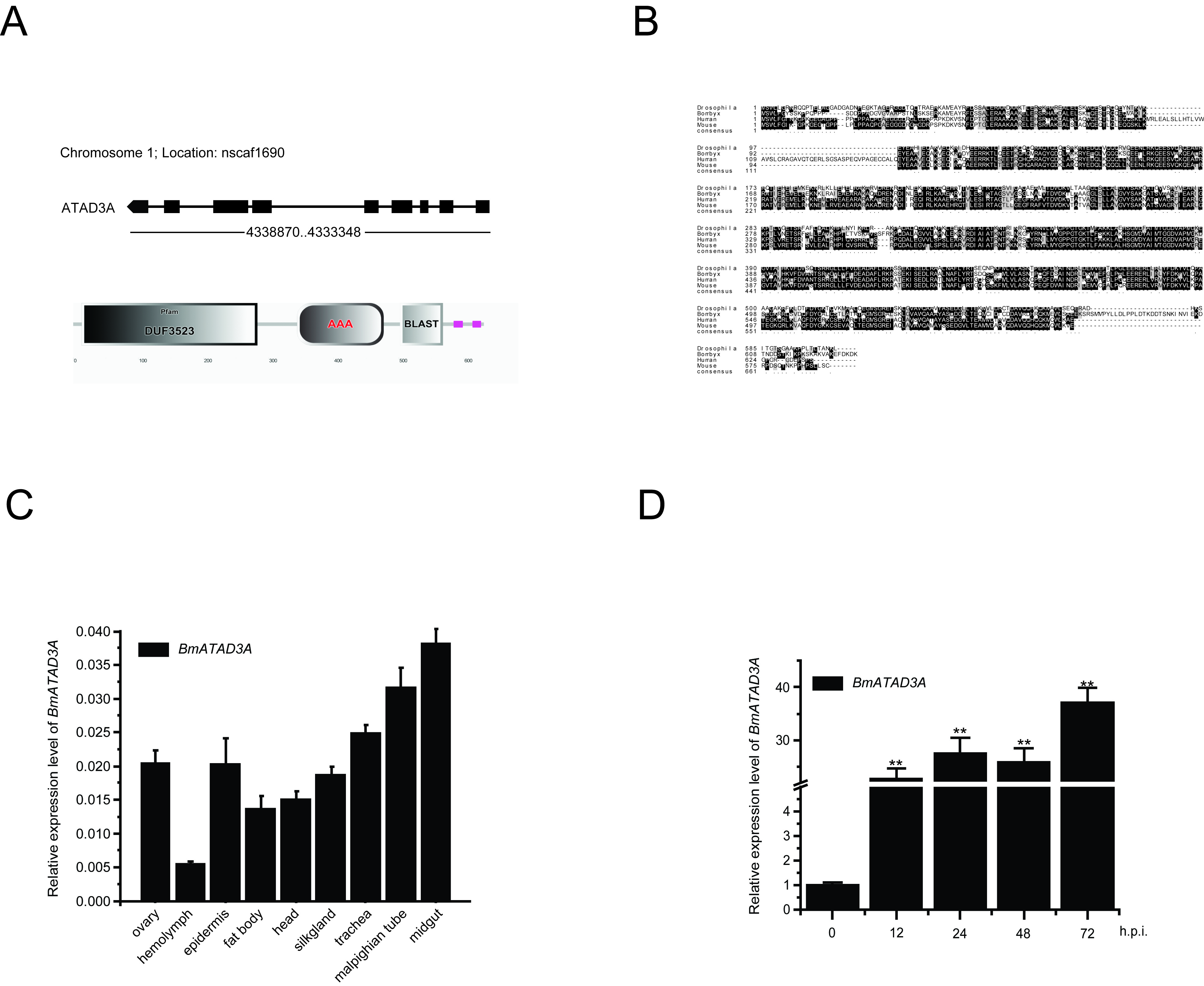


**S2 Fig. Characterization analysis of Bombyx mori ATAD3A protein.**

(A) Schematic representation of Bombyx mori ATAD3A protein. (B) Multiple sequences alignment of BmATAD3A. (C) Relative expression of BmATAD3A mRNA was detected in all tissues of Bombyx mori strains. The Bombyx mori larval tissues include ovary, hemolymph, epidermis, fat body, head, silkworm, trachea, malpighian tube, and midgut. (D) Relative expression level of BmATAD3A in BmNPV infection with BmN-SWU1 cells at MOI of 1. Error bars indicate standard deviations from the mean. NS, not significant, **P < 0.01.


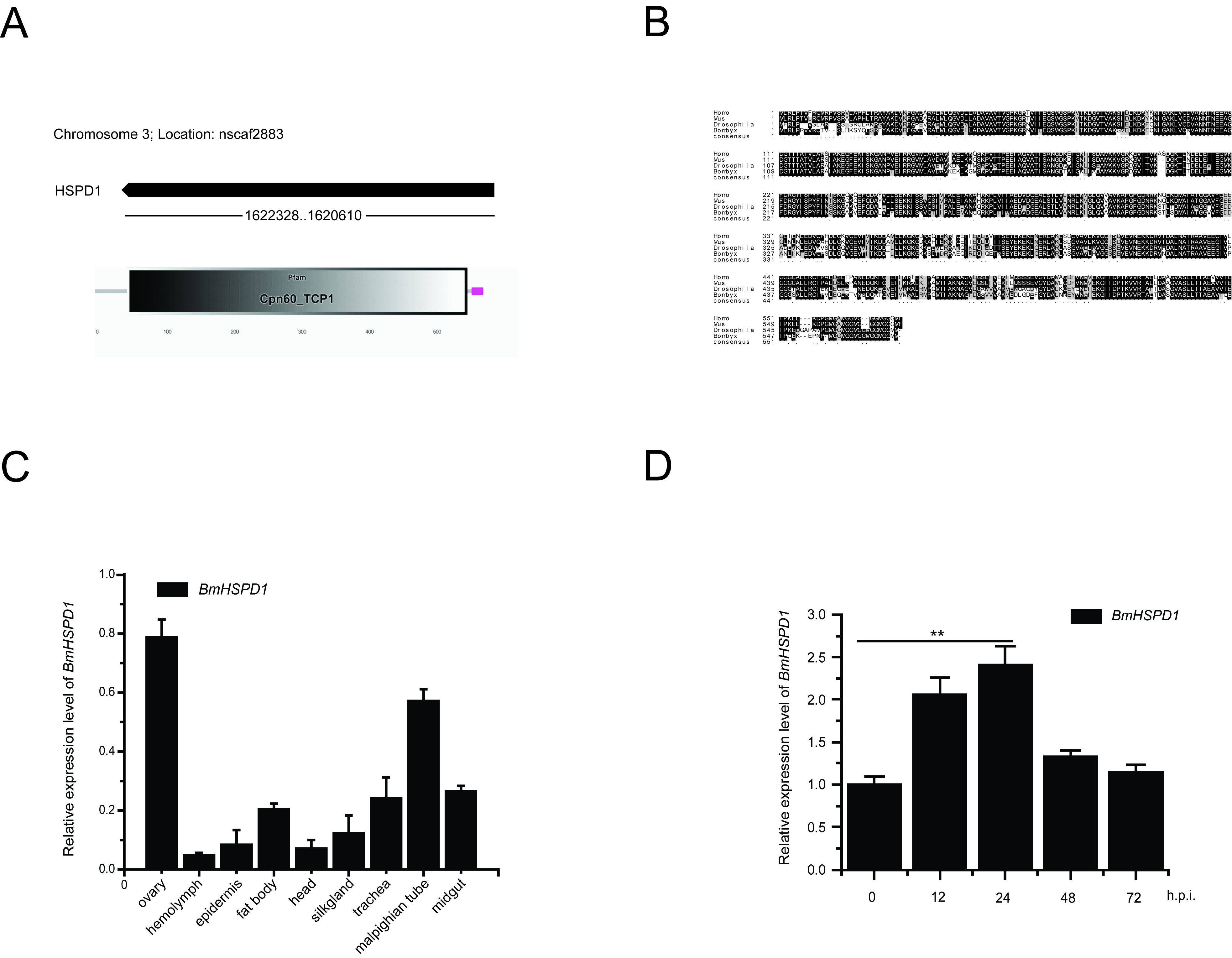


**S3 Fig. Characterization analysis of Bombyx mori HSPD1 protein.**

(A) Schematic representation of Bombyx mori HSPD1 protein. (B) Multiple sequences alignment of BmHSPD1. (C) Relative expression of BmHSPD1 mRNA was detected in all tissues of Bombyx mori strains. The Bombyx mori larval tissues include ovary, hemolymph, epidermis, fat body, head, silkworm, trachea, malpighian tube, and midgut. (D) Relative expression level of BmHSPD1 in BmNPV infection with BmN-SWU1 cells at MOI of 1. Error bars indicate standard deviations from the mean. NS, not significant, **P < 0.01.


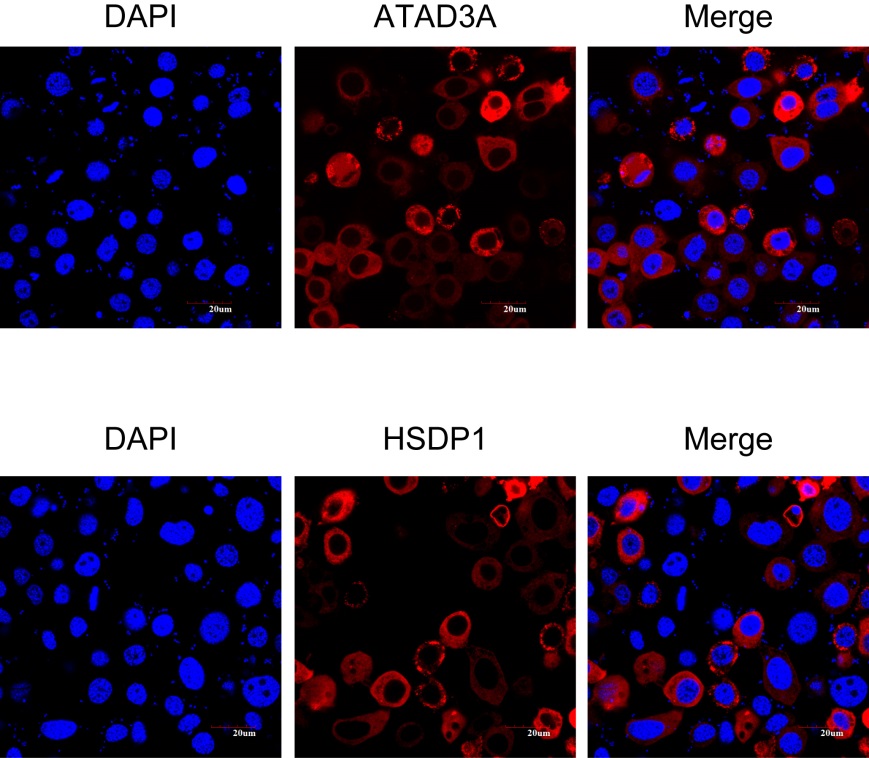


**S4 Fig. Screening of ATAD3A and HSPD1 stable expression cell lines.** The ATAD3A and HSPD1 expression cassette was transfected and screened in BmN-SWU1 cells using the Zeocin antibiotic. Red fluorescence represents the ATAD3A and HSPD1 expression cells and DAPI represents the cell nuclei.


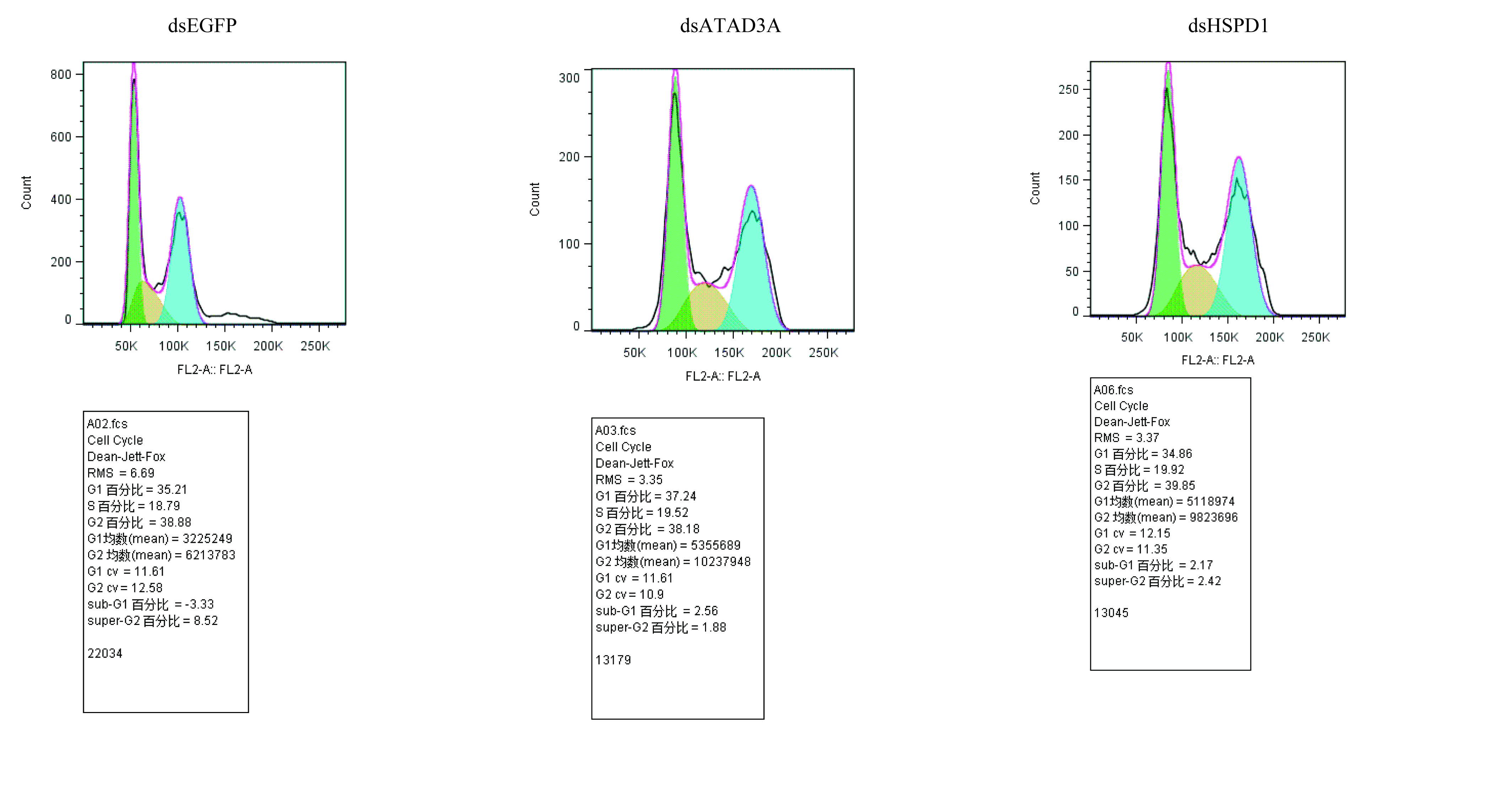


**S5 Fig. Cell cycle progression was measured by flow-cytometric analysis.** The dsEGFP, dsATAD3A and dsHSPD1 were transfected in BmN-SWU1 cells. Flow cytometry assessed the cell cycle progression of dsEGFP, dsATAD3A and dsHSPD1transfected cells, respectively.


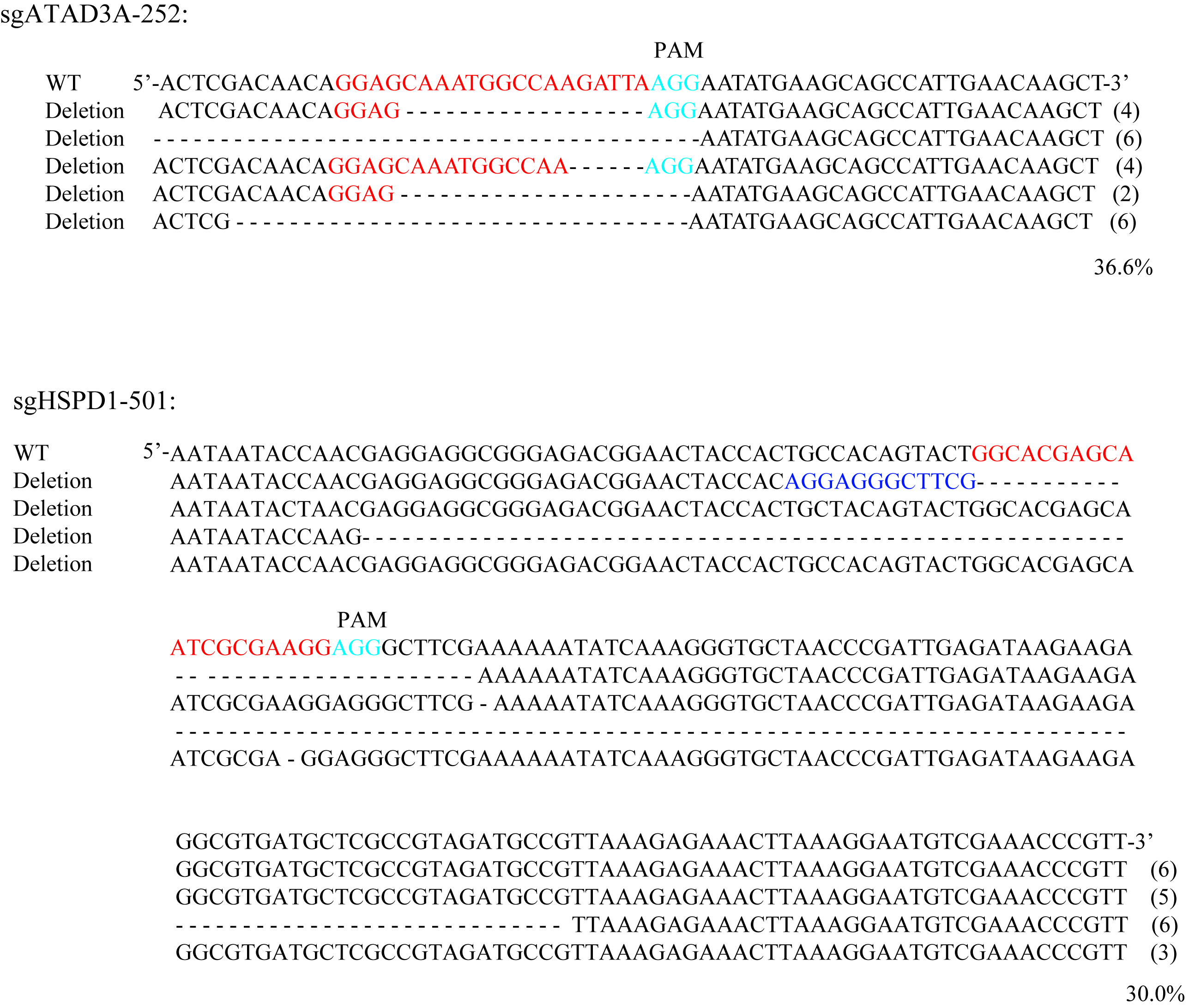


**S6 Fig. DNA sequence analysis of the CRISPR/Cas9 target sites in sgATA3A and sgHSPD1.** The WT sequence of *ATAD3A* and *HSPD1* is shown on top in bold, target sequence of sgRNA is indicated in red, and deletions are indicated by dashes. The numbers of mutations are marked accordingly on the right

**Supplementary Table 1.**

| Primer name | Primer sequence^*^ |
| --- | --- |
| RT-PCR primers | |
| RT-ATAD3A-F | 5' GGAGCGTAGTAGGTTCTGGTCTG 3' |
| RT-ATAD3A-R | 5' CAACCGATGTGGCTCCCT 3' |
| RT-HSPD1-F | 5' CGTCTACCTCGTGTTGTTCGTC 3' |
| RT-HSPD1-R | 5' CCCGCCTCCTCGTTAGTATTATT 3' |
| RT-SW22934-F | 5' TTCGTACTGGCTCTTCTCGT 3' |
| RT-SW22934-R | 5' CAAAGTTGATAGCAATTCCCT 3' |
| Co-IP primers | |
| ATAD3A-F | 5' CGGAATTCATGATGTCGTGGCTATTTGGGTA 3' |
| ATAD3A-R | 5' CCGCTCGAGTTATCTTTATCAAATTCCTTTGC 3' |
| Calmodulin-F | 5' GGGGTACCATGGCGGATCAGCTGAC 3' |
| Calmodulin-R | 5' GGACTAGTCTTCGACGTCATCATGGT 3' |
| HSC70-F | 5' GGACTAGTATGGCAAAAGCACCCG 3' |
| HSC70-R | 5' CCGCTCGAGATCGACCTCCTCGATGGT 3' |
| HSDP1-F | 5' CGCGGATCCATGTTGCGTCTACCTCGTGTT 3' |
| HSPD1-R | 5' CCGCTCGAGTCACATCATGCCTCCCATACCA 3' |
| HSP70-F | 5' CGGAATTCATGCCAGCTATCGGAATCG 3' |
| HSP70-R | 5' GCTCTAGAGTCGACTTCCTCAACTGTGGGT 3' |
| LEF11-F | 5' CGGGGTACCATGCCCCCCAAAAATT 3' |
| LEF11-R | 5' CGCGGATCCTTACAGGTCTTCTTCAG 3' |
| The target sequence in the *Bombyx mori* genome | |
| sgATAD3A-F | 5' AAGTGGAGCAAATGGCCAAGATTA 3' |
| sgATAD3A-R | 5' AAACTAATCTTGGCCATTTGCTCC 3' |
| sgHSPD1-F | 5' AAGTGGCACGAGCAATCGCGAAGG 3' |
| sgHSPD1-R | 5' AAACCCTTCGCGATTGCTCGTGCC 3' |
| sgMock-F | 5' AAGTGGAGGATGCATTAGCACAAC 3' |
| sgMock-R | 5' AAACGTTGTGCTAATGCATCCTCC 3' |
| GP41 primers | |
| GP41/F | 5' CCTATTCTGTGCTGGTGGTGG 3' |
| GP41/R | 5' ATGTTGATGTGCGGAAAGC 3' |
| Knockout detection primers | |
| HSPD1-F1 | 5' ATGTTGCGTCTACCTCGTGTT 3' |
| HSPD1-R953 | 5' GCTTCCTCTGTTGATTAGCCATTT 3' |
| ATAD3A-F23 | 5' GTTCAAAACCTCCACAACCTCC 3' |
| ATAD3A-R762 | 5' CAATAATGACAAACCCCCTGC 3' |
| dsRNA synthesis primers | |
| dsATAD3A-F | 5' TAATACGACTCACTATAGGGAGAAAGGGAGCCACATCGGTT 3' |
| dsATAD3A-R | 5' TAATACGACTCACTATAGGGAGATGCTGCTCTTAAATCCTCACTG 3' |
| dsHSPD1-F | 5' TAATACGACTCACTATAGGGAGAGTGCTAACCCGATTGAGATAAG 3' |
| dsHSPD1-R | 5' TAATACGACTCACTATAGGGAGAGGGTGGACTTGCGGTTATC 3' |
| dsEGFP-F | 5' TAATACGACTCACTATAGGGAGAACCATCTTCTTCAAGGACGACG 3' |
| dsEGFP-F | 5' TAATACGACTCACTATAGGGAGATGGGGGTGTTCTGCTGGTAG 3' |

^*^ (The restriction enzyme sites are marked in red).

**Supplementary Table 1. Sequences of primers used in this study.**
